# Selection Incentives in the Dutch Basic Health Insurance: To What Extent Does End-of-Life Spending Contribute to Predictable Profits and Losses for Selective Groups?

**DOI:** 10.1177/10775587221099731

**Published:** 2022-06-08

**Authors:** A. A. Withagen-Koster, R. C. van Kleef, F. Eijkenaar

**Affiliations:** 1 Erasmus University Rotterdam, The Netherlands

**Keywords:** health insurance, risk equalization, risk selection, survey data, end-of-life spending

## Abstract

Existing risk-equalization models in individual health insurance markets with premium-rate restrictions do not completely compensate insurers for predictable profits/losses, confronting insurers with risk selection incentives. To guide further improvement of risk-equalization models, it is important to obtain insight into the drivers of remaining predictable profits/losses. This article studies a specific potential driver: end-of-life spending (defined here as spending in the last 1–5 years of life). Using administrative (*N* = 16.9 m) and health survey (*N* = 384 k) data from the Netherlands, we examine the extent to which end-of-life spending contributes to predictable profits/losses for selective groups. We do so by simulating the predictable profits/losses for these groups with and without end-of-life spending while correcting for the overall spending difference between these two situations. Our main finding is that—even under a sophisticated risk-equalization model—end-of-life spending can contribute to predictable losses for specific chronic conditions.

## Introduction

Social health insurance markets with open enrollment and premium-rate restrictions typically rely on risk equalization to mitigate incentives for risk selection. An example of such a health insurance market can be found in the Netherlands, where regulated competition has been introduced to improve efficiency of the health care system while safeguarding individual affordability of basic coverage and accessibility of care. In this system, risk equalization is an important regulatory measure, which compensates insurers for predictable variation in health care spending among consumers (Van de Ven & Ellis, 2000; Van de Ven et al., 2013).

Although the Dutch risk-equalization model is one of the most sophisticated in the world, it does not completely compensate for predictable spending variation. Since insurers are not allowed to risk rate their premiums, this results in predictable profits and losses on specific subgroups of consumers. For example, recent research using diagnostic information from general practitioners has shown that Dutch insurers face a predictable loss of approximately 85 Euros per-person per year on the group with at least one chronic condition (approximately 52% of the population) and a predictable profit of 90 Euros per-person per year on the complementary group without a chronic condition (Van Kleef, Van Vliet, et al., 2018). This incentivizes insurers to attract healthy people (e.g., by selective marketing toward these groups) and to deter the chronically ill (e.g., by quality skimping). Such risk selection can distort both the efficiency of the health insurance market (e.g., via quality distortions) and jeopardize fairness of health care financing (e.g., when predictably profitable and predictably unprofitable people sort into different insurance plans, which threatens the level playing field and might result in selection-driven premium variation across plans) (Glazer & McGuire, 2000; Van de Ven et al., 2015; Van Kleef et al., 2019). Although the presence of risk selection is very difficult to demonstrate, Dutch health insurers have stated that they are reluctant to actively invest in health care for specific groups of chronically ill that remain to be undercompensated by the risk-equalization model (KPMG, 2014, 2020). This illustrates the importance of reducing the remaining predictable profits and losses. To guide further improvement of risk-equalization systems, it is important to obtain insight into the drivers of existing predictable profits and losses.

One possible factor that may contribute to the existing predictable profits and losses is end-of-life spending (defined here as spending in the last 1–5 years of life). On average, spending tends to be higher for individuals who are in the last phase of their life compared with those who are not (Polder et al., 2006; Van Vliet & Lamers, 1998). In addition, a significant share of total health care spending in a year can be attributed to end-of-life spending (Shmueli et al., 2010; Stooker et al., 2001). Moreover, Van Vliet and Lamers (1998) have shown that on average, health care spending of decedents starts to increase 6 years prior to death. To the extent that individuals who are in the last phase of their life are overrepresented in the subgroups used for evaluating selection incentives and the risk-equalization model does not adequately compensate for their high spending, end-of-life spending might significantly contribute to the remaining predictable profits and losses for these subgroups. Although the Dutch risk-equalization model includes many risk adjusters that compensate for high spending related to age, gender, and health, this model does not include a risk adjuster that explicitly flags people who are near the end of their life (Van Kleef, Eijkenaar, et al., 2018). Therefore, end-of-life spending might indeed contribute to remaining predictable profits/losses on selective groups. However, the end-of-life stage is likely to correlate with existing morbidity indicators in the risk-equalization model as a large share of the ex post spending on the deceased can be explained by ex ante spending on the sick (Einav et al., 2018). Therefore, the morbidity-based risk adjusters in the risk-equalization model may already compensate for a large share of end-of-life spending (Van Vliet & Lamers, 1998).

The aim of this article is to gain insight in the extent to which end-of-life spending contributes to existing group-level predictable profits and losses under sophisticated risk equalization. This insight will be helpful in guiding further improvements of the Dutch risk-equalization scheme. If we find a significant contribution of end-of-life spending to predictable profits and losses, improvement of the Dutch risk-equalization scheme should (also) focus on end-of-life spending, that is, by refining existing morbidity indicators or by applying some form of risk sharing. If we do not find a significant contribution, apparently other factors drive predictable profits and losses, and further research will be necessary to identify these factors. We use administrative data from the Netherlands (*N* ≈ 16.9 m) on both actual and predicted spending for 2013, and on whether someone has died during the period 2013–2017. We merge these data with health survey data from 2012 (*N* ≈ 384 k) and calculate predictable profits and losses in 2013 for selective subgroups of healthy and chronically ill individuals.

### New Contribution

An important difference between our article and previous papers focusing on end-of-life spending (Einav et al., 2018; Van Vliet & Lamers, 1998) is that rather than focusing on the predictability of end-of-life spending itself, we primarily focus on the contribution of end-of-life spending to selection incentives regarding selective groups of individuals. The underlying assumption is that insurers are likely and able to risk select based on health (e.g., yes/no chronic condition) rather than on yes/no being near the end of life. Although “health” can be known before the start of a contract period and thus be acted upon by insurers (e.g., in terms of marketing and design of health plans) and/or consumers (i.e., by choosing a certain plan), “being near the end of life” is hard to predict and thus difficult to act upon (Einav et al., 2018). To our knowledge, this is the first article that explicitly examines the extent to which end-of-life spending contributes to predictable profits and losses for subgroups that are vulnerable to risk selection. Since we use the actual risk-equalization data from the Netherlands, our empirical findings are directly relevant for that specific context. The international relevance of our article is to be found in the conclusion that—even under a sophisticated risk-equalization model as the one applied in the Netherlands—end-of-life spending can indeed contribute to the predictable losses for specific groups. What this contribution looks like in other settings is an empirical question and depends on specific contextual factors such as the benefits package, the characteristics of the population, and the features of the risk-equalization model in place. Our methodology can be useful for identifying the contribution of end-of-life spending to predictable profits and losses in other countries/settings.

This article is structured as follows. The next section describes the institutional setting, followed by the data and method used. Then, the results are reported. The last section summarizes and discusses the main findings.

### The Dutch Health Insurance System

The Dutch health insurance system consists of three components: a mandatory public insurance scheme for long-term care, a mandatory basic health insurance scheme providing coverage for curative care (e.g., primary care, pharmaceutical care, inpatient and outpatient hospital care and mental health care), and a voluntary supplementary health insurance covering services not covered by the two mandatory schemes. This article focuses on the basic health insurance system for curative care and all spending covered under that system, which operates on the basis of regulated competition among private insurers (Van Kleef, Eijkenaar, et al., 2018). In this system, competition among insurers is driven by a free consumer choice of insurance plan. Insurers have some flexibility regarding provider network and coverage of out-of-network spending, resulting in competition among providers of care. Relevant regulatory measures include an annual open enrollment requirement, community-rating per health plan, a standardized benefits package, an individual mandate to buy health insurance, and risk equalization.

The Dutch risk-equalization model of 2016, which is the focus of this article and has since 2016 undergone only relatively small changes, contains a broad set of sociodemographic risk adjusters (e.g., age interacted with gender, socioeconomic status and source of income interacted with age) as well as seven morbidity-based risk adjusters. Each risk adjuster consists of multiple risk classes, 162 in total. Payment weights for risk classes are estimated with a multivariate least-squares regression (of spending on risk classes) using data from a prior period (which has been made representative for payment year *t* in terms of benefits package, projected spending, and composition of the population). This results in a prediction model that allows for calculating individual-level spending in Euros, which forms the basis for the risk-equalization payments. More specifically, the risk-equalization payment for enrollee i is calculated as i’s predicted spending minus a fixed amount Y. The value for Y is determined by the Minister of Health and reflects the amount of spending that has to be financed via the out-of-pocket premium. The community-rated out-of-pocket premium reflects Y as well as the relative (in)efficiency of insurers, thereby creating price competition. From the perspective of insurers, part of their revenues (about 50%) comes from the risk-equalization fund (which itself is financed by ear-marked income-related contributions) and the other part (also 50% in total) comes from out-of-pocket premiums. Risk-equalization transfers are executed by the Dutch Healthcare Institute.

The morbidity-based risk adjusters in the Dutch model include Pharmacy-based Cost Groups (PCGs), Diagnosis-based Cost Groups (DCGs), Multiple-year High-Cost Groups (MHCGs), Durable Medical Equipment Cost Groups (DMECGs), physiotherapy spending in the prior year, geriatric rehabilitation care spending in the prior year, and home care spending in the prior year. Each of these morbidity adjusters is “prospective,” which means that these indicators are based on information from a prior period. For example, the PCG-adjuster consists of 33 classes based on individuals’ medication use in the prior year. To be flagged by a PCG, individuals must pass a predetermined defined daily dose (DDD) threshold of the relevant medication. The DCG-adjuster comprises 15 classes in which individuals can be classified based on specific diagnoses from inpatient and outpatient hospital treatment in the prior year. The MHCG-adjuster contains seven classes based on the level of spending for curative somatic care in the three prior years. The assumption is that individuals with multiple-year high costs most likely suffer from a chronic condition. Individuals can be flagged by one of the four classes of the DMECG-adjuster based on the use of specific durable medical equipment in the prior year. The remaining three morbidity-based risk adjusters are all based on prior-year spending on specific types of care, that is, physiotherapy, geriatric rehabilitation care, and home care. For more information about the Dutch risk-equalization system, we refer to Van Kleef, Eijkenaar, et al. (2018).

## Data and Method

### Data

To examine the contribution of end-of-life spending to group-level profits and losses, we merged three data sets using an anonymized individual-level identification key. The first data set includes administrative data on individual-level spending and risk adjusters for all Dutch citizens with a basic health insurance in 2013 (*N* ≈ 16.9 million). These data allow us to replicate the Dutch risk-equalization model.

The second data set contains information on whether someone has died in the period 2013–2017 and, if so, in which year. This information enables us to identify people in our 2013 data who are near the end of their life. Given our data, we take into account five definitions of “being near the end of life”: deceased within 1 year (i.e., died in 2013), deceased within 2 years (i.e., died in 2013–2014), deceased within 3 years (i.e., died in 2013–2015), deceased within 4 years (i.e., died in 2013–2016), and deceased within 5 years (i.e., died in 2013–2017).^1^

The third data set comes from a health survey conducted in 2012 (*N* ≈ 384 k). This data set contains information on self-reported chronic conditions by individuals aged 19 years or older on September 1, 2012 (Public Health Monitor, 2016). We use these data to define subgroups that are potential targets of risk selection by insurers (e.g., by selective advertising and insurance plan design). These groups have been extensively analyzed in previous studies and are considered relevant when it comes to the evaluation of the Dutch risk-equalization model in terms of selection incentives (Van Kleef et al., 2013a, 2013b, 2017, 2020; Withagen-Koster et al., 2020).

Before conducting the evaluation of predictable profits/losses, we rebalanced the health survey sample using a raking procedure. Although the unbalanced sample is already quite representative for the population (Van Kleef et al., 2017), this procedure enabled us to further improve upon this. The raking procedure is an iterative process that generates a weight for every record in our data set using a set of key variables that are present in both the survey sample and the total population. Application of these weights ensures that the frequencies of these variables in the sample are similar to those in the population (Battaglia et al., 2009; Izrael et al., 2000). The set of key variables includes all risk-adjuster classes of the Dutch risk-equalization model of 2016, as well as 18 quantiles of mean curative somatic spending and a proxy for whether someone had died in 2013.^2^ For a more detailed description of the rebalancing procedure as well as results on the sample’s representativeness before and after rebalancing, see Withagen-Koster et al. (2020).

### Method

To determine the contribution of end-of-life spending to predictable profits and losses for selective subgroups, we performed a simulation that consisted of seven steps. First, we merged the administrative data on 2013 spending and risk-adjuster flags with the data on the year of death (indicating who in the administrative data of 2013 died in the period 2013–2017). In an explorative analysis, we examined the characteristics in 2013 of those who died in the period 2013–2017.

Second, we used the health survey data to define 25 selective subgroups that are potentially vulnerable to risk selection. More specifically, we identified 19 specific chronic conditions that individuals in 2012 could report to have suffered from in the past 12 months, and four chronic conditions that individuals in 2012 could report to have suffered from ever in the past. In addition, we constructed two more general groups based on yes/no self-reported chronic condition (ever or in the past 12 months).

Third, as a baseline, we estimated the actual Dutch risk-equalization model of 2016 on the total population (*N* = 16.9 m). This baseline model will be referred to as M1. Under M1, the payment an insurer receives for a certain enrollee equals the predicted spending for that enrollee generated by the risk-equalization model.

Fourth, we supplemented M1 with a 100% cost-based compensation for people near the end of their life. We refer to this model as M2. More specifically, we applied the Dutch risk-equalization model of 2016 to the total population excluding those near the end of their life. For individuals in the group on which the model was estimated, payments equal predicted spending generated by this model. For excluded individuals (i.e., those near end of life), we set the payment equal to actual spending (i.e., a 100% cost-based compensation). This scenario essentially implies “carving out” end-of-life spending. Compared with M1, M2 is expected to reduce predictable profits and losses on the subgroups of Step 2, because it is likely these subgroups include some decedents with end-of-life spending. This reduction, however, is not necessarily (fully) attributable to end-of-life spending and can thus not be interpreted as such. The reason is that any “carving out” of spending is likely to reduce predictable profits and losses. For example: in the extreme situation in which all spending would be “carved out” (i.e., 100% cost-based compensation for all individuals in the population), predictable profits and losses would drop to zero.

To determine whether the reduction in predictable profits and losses under M2 is indeed attributable to end-of-life spending, we cannot compare M2 with M1 as this would be an apples-to-oranges comparison due to the difference in overall mean spending. Therefore, we defined a third model (M3). This is the fifth step of our simulation analysis. Whereas M2 supplements the baseline model (M1) with a 100% cost-based compensation for people near the end of life, M3 supplements M1 with a form of proportional cost-based compensation for the entire population with positive spending. Specifically, under M3—which functions as a counterfactual for M2—we carve out the same proportion of total spending as under M2, but then across the entire population instead of only for certain groups. While under M2 we carve out 100% of spending for individuals near the end of life, under M3 we carve out X% of spending for all individuals, with X being the share of end-of-life spending in total spending (defined as the sum of spending for people near the end of their life divided by the sum of spending in the total population. See also the last column of Table 2).

Sixth, for each of the 25 subgroups identified in Step 2 and for each of the models M1 to M3 described in Steps 3 to 5, we calculated the mean per-person profit/loss as the mean payment that insurers receive for a group minus the mean spending for that group.^3^

Finally, for each group we tested whether the differences in the profits/losses between M2 and M3 are statistically significant using a paired *t*-test. If the profit/loss for a certain group under M2 (with spending being carved out for those near end of life) is statistically significantly different from that under M3 (with the same proportion of total spending being carved out as under M2, but then in the form of a percentage of spending for each and every individual in the population), we can conclude that end-of-life spending contributes to the existing profit/loss for that group under the baseline scenario (M1).

We repeated Steps 4 to 7 for each of the five definitions of “being near the end of life” described in the “Data” section.

## Results

This section presents the results of our simulations. First, some descriptive statistics of the administrative and health survey data are presented, followed by the characteristics of the deceased. Next, we provide information on the proportion of the population near the end of life in our subgroups. Finally, we quantify the contribution of end-of-life spending to the predictable profits and losses on these groups.

### Descriptive Statistics

Table 1 shows descriptive statistics of the unbalanced and rebalanced survey sample and the population in the administrative data for individuals aged 19 years and older on September 1, 2012. After rebalancing, the statistics for the sample match those in the population very well.

**Table 1.** Mean Curative Somatic Spending and Population Frequencies in 2013 for Selected Risk-Adjuster Variables in the Unbalanced and Rebalanced Survey Sample and the in Terms of Age Corresponding Population in the Administrative Data (19 Years or Older on September 1, 2012).

	Survey sample (unbalanced)	Survey sample (rebalanced)	Population
*N*	384,004	384,004	12,774,886
Mean spending in Euros (*SD*)	3,216 (8,909)	2,460 (7,793)	2,460 (8,016)
Mean predicted spending (*SD*)	3,247 (5,179)	2,460 (4,558)	2,460 (4,554)
Man, 19–34 years	5.6%	11.8%	11.8%
Man, 35–44 years	4.8%	8.7%	8.7%
Man, 45–54 years	6.6%	9.8%	9.8%
Man, 55–64 years	7.6%	8.4%	8.4%
Man, 65 years and older	20.7%	10.1%	10.1%
Woman, 19–34 years	8.0%	11.8%	11.8%
Woman, 35–44 years	6.5%	8.8%	8.8%
Woman, 45–54 years	8.4%	9.8%	9.8%
Woman, 55–64 years	8.5%	8.4%	8.4%
Woman, 65 years and older	23.4%	12.4%	12.4%
PCGs	33.1%	24.1%	24.1%
DCGs	16.2%	11.5%	11.5%
MHCGs	10.4%	7.1%	7.1%
DMECGs	1.5%	1.1%	1.1%
Physiotherapy spending in the previous year	3.8%	2.6%	2.6%
Home care spending in the previous year	3.7%	2.6%	2.6%
Geriatric rehabilitation care spending in the previous year	0.3%	0.3%	0.3%

*Note.* PCGs = Pharmacy-based Cost Groups; DCGs = Diagnosis-based Cost Groups; MHCGs = Multiple-year High-Cost Groups; DMECGs = Durable Medical Equipment Cost Groups.

Table 2 provides descriptive information on our five definitions of being near the end of life. Of the total population in 2013, 0.8% died in 2013. We find similar annual death rates for later years, leading to 4.3% of the 2013 population having died in the period 2013–2017. For those who died in 2013, spending amounts to 5.3% of total spending. This percentage increases to 20.1% after inclusion of those who died in the 4 years thereafter. Note that the percentages in the last column are used as a basis for the proportional cost-based compensations under M3 (i.e., they represent the proportion “X” as described in Step 5 in the “Method” section).

**Table 2.** Deceased as Percentage of Total Population in 2013 and End-of-Life Spending as Percentage of Total Spending in 2013, for Five Definitions of Being Near the End of Life.

Definitions of being near end of life	Deceased as % to total population in 2013	Spending of people near end of life as % of total spending in 2013
Deceased in 2013	0.8%	5.3%
Deceased in 2013–2014	1.6%	10.5%
Deceased in 2013–2015	2.5%	14.2%
Deceased in 2013–2016	3.4%	17.3%
Deceased in 2013–2017	4.3%	20.1%

### Characteristics in Year *t* of Those Being Near the End of Life (Survey Sample)

This section presents characteristics in 2013 for the following groups (conditional on the survey sample): those who died in 2013, those who died in 2014, those who died in 2015, those who died in 2016 and those who died in 2017. For each of these five groups, Table 3 shows the mean actual and predicted spending, the mean profit/loss, the percentage flagged by a morbidity-based risk adjuster, and the percentage with a self-reported chronic condition. For those who died in the period 2013-2017, average spending in 2013 is higher than for the average person in the sample. The same is true for predicted spending in 2013, indicating that the risk-equalization model explains some of the spending variation between people who are near the end of their life and those who are not. This is supported by the high percentage of individuals flagged by at least one morbidity-based risk adjuster.

**Table 3.** Mean (Predicted) Spending, Mean Ex Post Profit/Loss, Percentage Flagged by at least One Morbidity-Based Risk-Adjuster, and Percentage With (at least) One or More Self-Reported Chronic Condition(s) in 2013, for the Total Survey Sample and for Those Who Died in the Period 2013–2017 in That Sample.

		Died in					
		Total sample	2013	2014	2015	2016	2017
Mean spending in Euros (*SD*)		2,460 (7,793)	32,408 (42,498)	17,436 (25,854)	11,388 (17,421)	9,480 (16,391)	8,684 (14,774)
Mean predicted spending in Euros (*SD*)		2,460 (4,558)	12,850 (12,802)	11,496 (13,306)	9,210 (11,329)	8,759 (9,689)	8,244 (10,256)
Mean ex post profit/loss in Euros		0	−19,558	−5,940	−2,178	−721	−440
Flagged by at least one morbidity-based risk adjuster included in the risk-equalization model		31.0%	84.3%	79.2%	77.8%	76.1%	74.7%
At least one self-reported chronic condition (ever or in past 12 months)	Yes	53.3%	80.7%	72.9%	74.2%	74.5%	74.2%
	No	34.3%	6.2%	8.1%	10.8%	9.0%	9.2%
	Missing	12.4%	13.2%	19.0%	14.9%	16.6%	16.6%
Diabetes (ever)		5.8%	17.8%	16.3%	17.5%	18.5%	17.2%
Stroke (ever)		2.9%	15.9%	14.8%	14.9%	10.7%	11.0%
Heart attack (ever)		3.0%	13.6%	14.1%	14.9%	13.1%	12.9%
Cancer (ever)		6.5%	34.3%	26.6%	25.8%	21.2%	18.4%
Number of self-reported chronic conditions (ever or in past 12 months)	One	24.6%	20.1%	24.1%	25.9%	25.1%	26.2%
	Two	13.1%	19.3%	20.3%	18.8%	19.8%	19.4%
	Three	6.8%	16.1%	14.4%	13.7%	14.7%	14.6%
	Four or more	8.7%	37.4%	31.2%	28.8%	29.7%	28.8%

*Note.* The “number of self-reported conditions” is based on the conditions respondents could report to have suffered from ever in the past (i.e., the four conditions listed) as well as the conditions respondent could report to have suffered from in the past 12 months (listed in Table B1)

Table 3 also shows a significant gap between predicted and actual spending for all groups. This gap is largest for those who are within 1 year from death (loss of 19,558 Euros) and smallest for those who are 3 to 4 years from death (loss of 721 and 440, respectively). On average, spending in 2013 for those who died between 2013 and 2017 equals 15,879 Euros while predicted spending in 2013 for this group equals 10,112 Euros, implying an ex post loss of 5,767 Euros in 2013. Note that this ex post loss is not necessarily predictable ex ante.^4^ However, to the extent that this ex post loss is concentrated in our subgroups of interest, it might contribute to the predictable losses on these groups.

Table 3 further shows that people near the end of life are much more likely to be flagged by at least one morbidity-based risk adjuster than the average person in the sample (31.0%). The percentage with at least one morbidity flag is somewhat higher for those who are less than 1 year from death (84.3%) than those who are more than 1 year from death (between 79.2% and 74.7%). The percentage of individuals with at least one self-reported chronic condition is also much higher among those near the end of life (ranging from 74.2% to 80.7%, depending on the definition) than in the sample (53.3%). Moreover, as becomes clear from the bottom three rows, people who are near the end of their life suffer more often from multimorbidity. This makes sense since those who are very ill are more likely to die. This is also what we see when we analyze the relative frequency of decedents within each of the morbidity-based risk classes (as described in “The Dutch health insurance system” section). When we look at the relative frequency of those who died in 2013, we find the highest values for DCG 11 (17%) which includes people with cancer and spina bifida, for DCG 13 (11%) which includes people with aplastic anemia and users of home ventilation, for PCG 17 (10%) and 29 (13%) for Parkinson’s disease and cancer medication, respectively, and finally, for the highest class of the home care risk adjuster (11%), which includes individuals with spending in the top 0.25% for home care in the prior year. When we look at the relative frequency within each of the morbidity-based risk adjusters conditional on the group of people who died in the period 2013–2017, we find the highest values for the highest three classes of the home care risk adjuster (46%–58%) which includes individuals with spending in the top 1.5%, top 0.5%, and top 0.25% of home care spending in the prior year, DCG 15 for people requiring renal dialysis and PCG 26 for kidney disease (both 46%).

### Share of Deceased in Subgroups From the Health Survey

**Figure 1.**
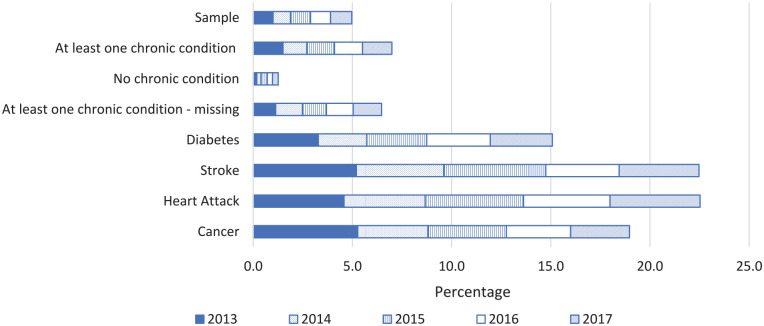
Decedents in 2013, 2014, 2015, 2016, and 2017 as a Percentage of the Total Survey Sample in 2013 and of Specific Subgroups Identifiable in That Sample. *Note.* “Sample” refers to the whole health survey sample. The groups “at least one chronic condition,” “no chronic condition,” and “at least one chronic condition-missing” are determined based on chronic conditions respondents could report to have ever suffered from or in the past 12 months. The specific groups “diabetes,” “stroke,” “heart attack,” and “cancer” refer to chronic conditions people could report to have ever suffered from in the past.

The previous section has shown that individuals with a morbidity-based risk adjuster (i.e., suffering from a chronic condition according to the risk-equalization model) are overrepresented among those near the end of life. But to what extent are individuals near the end of life overrepresented among those who reported to suffer from a chronic condition in the health survey? Figure 1 answers this question for a selection of subgroups identifiable in the survey sample. As can be seen, 5 percent of the total sample has died after five years (2013-2017). In line with expectations, this percentage is indeed higher in the group “at least one chronic condition” (7%) and lower for the group “no chronic condition” (1.3%). The bottom four groups are specific chronic conditions that individuals could report to have ever suffered from in the past. The group “cancer” has the highest death rate in the first year (5.3%), while after five years the cumulated rate is highest in the groups “stroke” and “heart attack” (both 22.5%).

### Contribution of End-of-Life Spending to Group-Level Profits and Losses

**Figure 2.**
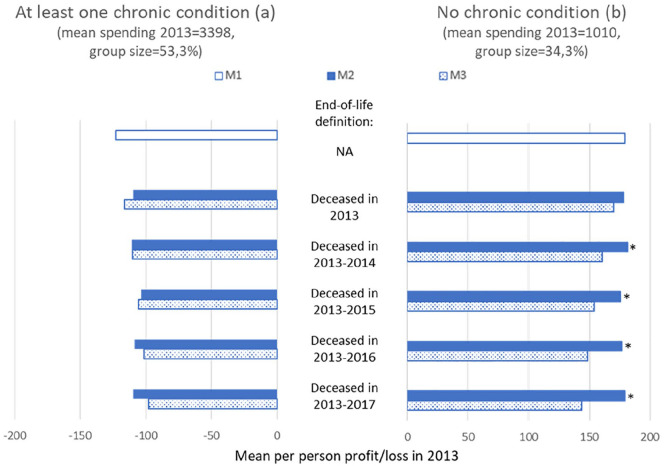
Mean Per-Person Profit/Loss in 2013 Under M1 to M3 for Five Definitions of Being Near the End of Life for the Group Who in 2012 Reported to Suffer From at least One Chronic Condition and the Group Who in 2012 Reported to Suffer From No Chronic Condition *Note.* Due to the group with missing values for yes/no self-reported chronic condition, the groups in panel a and b do not add up to 100%. See Appendix A for the results on the group with missing values. M1 is the Dutch risk-equalization model 2016, M2 is M1 supplemented with 100% cost-based compensation for people who are near the end of life, and M3 is M1 supplemented with a proportional cost-based compensation using the percentages in the right column of Table 2. NA means not applicable. An asterisk (*) next to a set of bars indicates a statistically significant (*p* < .05) difference between the profit/loss under M2 versus M3.

This section presents the mean per-person profits/losses by subgroup under the three models (M1–M3) for our five definitions for “end of life.” M1 replicates the Dutch risk-equalization model of 2016, M2 is M1 supplemented with 100% cost-based compensation for (spending of) people who are near the end of their life, and M3 is M1 supplemented with a proportional cost-based compensation using the percentages in the right column of Table 2. Figure 2 shows the results for these models for two groups: those who in 2012 (*t* − 1) reported to have suffered from at least one chronic condition (ever or in the past 12 months, panel a) and those who reported not to have suffered from a chronic condition (panel b). As expected, under M2, the predictable loss on the group with a chronic condition is lower than under M1, for each definition of being near the end of life. For end-of-life spending to have really contributed to the predictable loss on this group, the loss under M2 (filled bars) must be statistically significantly lower than the value under M3 (scattered bars). As this is not the case for any of the five definitions of end-of-life spending, we cannot conclude that end-of-life spending contributes to the predictable loss on this group. For the group without a chronic condition, we find that the predictable profit does not really reduce under M2 compared with M1. In an additional analysis (not shown here), we found that—for this specific group—actual spending and predicted spending roughly reduce to the same extent, thereby leaving the size of the gap (i.e., the predictable profit) intact. As a result, the predictable profit under M2 is higher than under M3. (Note that under M3 both actual spending and predicted spending decrease proportionally compared with M1, implying that because this groups is overpaid, the absolute reduction is higher for predicted spending than for actual spending. Consequently, the predictable profit for this group decreases under M3 compared with M1.)

We also examined the mean per-person profit/loss under models M1–M3 for the group of individuals for whom the information “yes/no self-reported chronic condition” is missing (see Appendix A). The per-person profit under M1 is much closer to zero compared with the groups “at least one self-reported chronic condition” and “no self-reported chronic condition,” indicating that the group for which this information is missing is not a selective group. For this group, we also do not find a statistically significant difference between M2 and M3 for any of the five definitions of end-of-life spending indicating that end-of-life spending does not contribute to the remaining profits.

**Figure 3.**
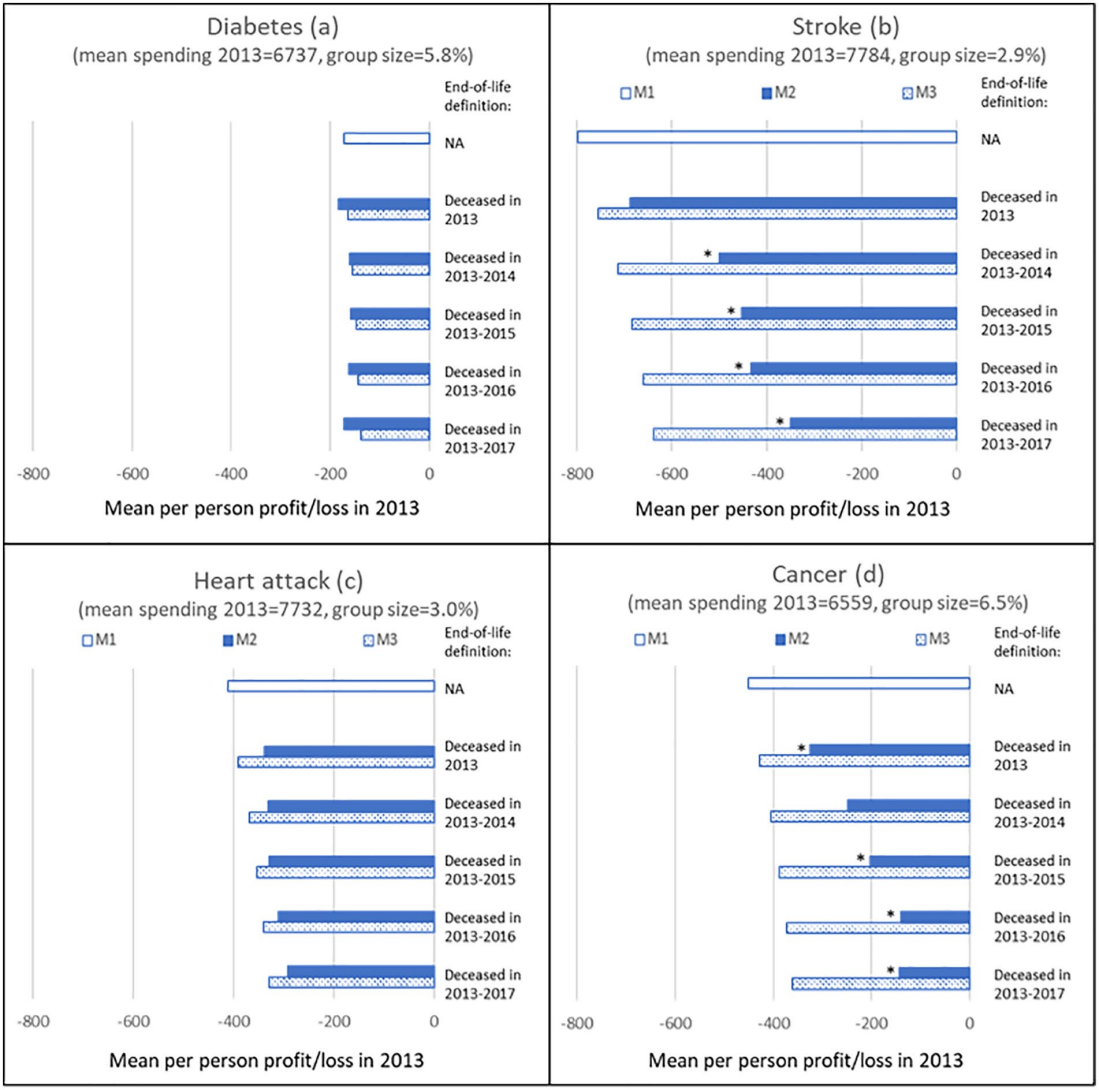
Mean Per-Person Loss in 2013 Under M1 to M3 for Five Definitions of Being Near the End of Life for Individuals Who in 2012 Reported to Have Ever Suffered From Diabetes, Stroke, Heart Attack, or Cancer. *Note.* M1 is the Dutch risk-equalization model 2016, M2 is M1 supplemented with 100% cost-based compensation for people who are near the end of life, and M3 is M1 supplemented with a proportional cost-based compensation using the percentages in the right column of Table 2. NA means not applicable. An asterisk (*) next to a set of bars indicates a statistically significant (*p* < .05) difference between the profit/loss under M2 versus M3.

Figure 3 shows results for individuals who reported to have ever suffered from diabetes (panel a), stroke (panel b), heart attack (panel c), or cancer (panel d). For diabetes, M2 results in a slightly higher predictable loss than M1 under the definition “deceased in 2013.” With the expansion of the end-of-life definition, a small decrease followed by a small increase can be observed, but none of these results are statistically significantly different from those under M3.

For stroke, the loss under M2 is smaller than under M1 for all definitions of end of life (panel b). Except for the definition “deceased in 2013,” the loss is also statistically significantly lower than under M3, suggesting that end-of-life spending contributes to this loss. Compared with M3, the loss is 30% to 45% lower, depending on the definition of being near end of life.

Panel c shows that for the group “heart attack,” the predictable loss under M2 keeps declining as the end-of-life definition encompasses more years before death. However, there is no statistically significant difference with the losses under M3 for any of the definitions.

Finally, panel d shows a declining loss for the group “cancer” under M2 compared with M1 for all definitions. The loss under M2 is statistically significantly lower than that under M3 for al definitions of end of life. Compared with M3, this loss is approximately 40% to 60% lower, depending on the definition.

Appendix B shows the mean per-person profit/loss under the three models M1 to M3 and five definitions of being near end of life for 19 specific chronic conditions respondents could report to have suffered from in the past 12 months (instead of ever in the past, like in Figure 3). For 15 groups, the predictable losses under M2 are not significantly different from those under M3 for any of the definitions of being near the end of life. The four exceptions are the groups “cancer,” “blood pressure,” “blood vessels,” and “joint inflammation.” In general, for these groups, the difference in predictable loss between M3 and M2 is more likely to be statistically significant for the more comprehensive end-of-life definitions.

## Discussion

In this article, we determined the contribution of end-of-life spending to predictable profits and losses that insurers face for selective subgroups in the Dutch basic health insurance. In line with prior research (Einav et al., 2018; Van Vliet & Lamers, 1998), our descriptive analyses show that decedents have higher spending on average and that spending is already higher up to 5 years prior to death. In addition, those who will die within 5 years are more likely to be flagged by a morbidity-based risk adjuster included in the risk-equalization model. Our descriptive analyses further show that on average people near the end of life do suffer more often not only from a chronic disease but also from multimorbidity.

To determine the contribution of end-of-life spending to predictable profits and losses on groups of interest, we simulated these profits and losses with and without end-of-life spending, while correcting for the overall difference in mean spending between these two situations. Our results show that end-of-life spending contributes to the predictable profits and losses for some groups, but not for others. Accounting for end-of-life spending significantly reduced the predictable losses for some groups, like individuals who indicated to have suffered from stroke (ever) or cancer (ever or in the past year), or a condition of the blood vessels (in the past year). Individuals with these chronic conditions might not always be flagged as such by the risk-equalization model, for instance, because they do not cross the defined daily dose (DDD) threshold to be flagged by a PCG (although they might use the relevant medication), and/or because they did not have a hospital treatment in the prior year (and thus no DCG flag). This could explain why we find an effect for these groups.

To reduce selection incentives for these specific groups, better incorporating “being near the end of life” in the (risk-adjusted) compensation would reduce the predictable losses. One possibility to do so is to refine existing morbidity-based risk adjusters and/or to add new risk adjusters. In this respect, explicitly accounting for the multiple year character of the aftermath of a disease could prove helpful. For instance, individuals who had a stroke in a certain year do not only have above-average spending in that and the next year but probably also in the years thereafter. This high spending in later years is unlikely to be fully captured by the risk-equalization model because morbidity flags tend to be based on utilization in the previous year. Another option is to use a form of cost-based compensation, for example, the form applied in this article (i.e., an ex post compensation of actual spending by people who—in retrospect—turned out to be near end-of-life) or some form of outlier risk sharing (i.e., a compensation for X% of actual individual-level spending above a predefined threshold). Application of such cost-based compensations, however, comes at a cost: although they can mitigate predictable profits and losses, they also reduce insurers’ incentives for cost control. To guide the choice of policy measure(s) to better compensate for end-of-life spending, we recommend conducting a more in-depth analysis of specific health care of people near the end of life (which was not possible with the data available for this study). This might provide valuable insights in the type of ex ante information available for better identification of those near end of life.

We did not find a statistically significant contribution of end-of-life spending to the predictable profits and losses for all subgroups of chronically ill. For some groups, like heart attack, this might be considered surprising. One explanation for this is that carving out end-of-life spending can have a direct and an indirect effect, which for some groups might balance each other out. The direct effect is that carving out end-of-life spending lowers overall spending within subgroups, reducing profits/losses. The indirect effect is that the payment weights of the risk adjusters in the risk-equalization model change. More specifically, carving out end-of-life spending lowers the payment weights for especially the morbidity-based risk adjusters, resulting in lower risk-adjusted payments. Another possible explanation for the absence of a statistically significant contribution of end-of-life spending for some groups, like diabetes, is that most of the chronically ill individuals in these groups are also flagged as such by the risk-equalization model (and the model also adequately captures the aftermath of a disease).

Our study comes with at least three limitations. First, our findings based on the health survey data are conditional on the adult population of 19 years and older. Moreover, the survey data are from a sample of the population and might suffer from some observation bias. We improved the representativeness of the survey as much as possible by applying a raking procedure. Nonetheless, some observation bias might remain. The third limitation regards the generalizability of our findings beyond the context of the Dutch health insurance system. The international relevance of our study can be found in the conclusion that—even under sophisticated risk equalization—end-of-life spending can significantly contribute to predictable profits and losses for selective groups. The groups, for which this is true, however, will vary across health care systems and depend on contextual factors such as the benefits package, features of the risk-equalization model, and characteristics of the relevant population. For example, the risk-equalization model used in Medicare Advantage can also be considered sophisticated, but unlike the Dutch model relies on concurrent rather than prospective morbidity flags. Moreover, the two models use different types of risk adjusters. Furthermore, Medicare Advantage pertains to the population of 65 years and older, whereas the Dutch basic health insurance covers the entire population. Therefore, the exact contribution of end-of-life spending to predictable profits and losses in Medicare Advantage and other systems might differ from that in the Dutch setting and remains an empirical question. The methodology used in this article could help to answer this question for health care systems in other countries.

Regardless of whether end-of-life spending is accounted for, we find significant predictable profits and losses for all subgroups studied. This suggests that there are other factors that drive these profits and losses. To be able to further mitigate incentives for risk selection, it is important to identify these factors. Potential starting points could be analyses of the heterogeneity of the groups flagged by the morbidity-based risk adjusters in the risk-equalization model (i.e., how selective are these groups?), and of the reason(s) for why chronically ill individuals are not always identified as such by the risk-equalization model. Another option to further mitigate incentives for risk selection is using risk adjusters that are based on information from the current year instead of from a prior period. An example of a model using such concurrent risk adjusters can be found in the marketplaces in the United States. The advantage of a concurrent model over a prospective model as currently used in the Netherlands is a better model fit. However, a prospective model is better able to maintain and stimulate cost control incentives.

In this article, we assumed that risk selection will most likely take place on the basis of “health” (e.g., yes/no chronic condition). An interesting question is whether risk selection might also take place based on yes/no being near end of life. For this to be true, three preconditions must be met: (a) being near the end of life comes with a profit/loss for the insurer, (b) being near the end of life must be predictable to some extent, and (c) insurers must be able to target this group with specific actions (Ellis & McGuire, 2007; Van Kleef et al., 2020). Regarding the first condition, our results indicate a loss of approximately 5,700 Euros for individuals who will die in the next 4 to 5 years, implying that this precondition is met. Regarding the predictability of being near end of life, previous research has clearly shown that it is hard to predict who will die in the upcoming year (Einav et al., 2018; Van Vliet & Lamers, 1998). However, this might be different for those who will die in the next 4 or 5 years. Given the extensive medical information that has become increasingly available for specific conditions (e.g., cancer) and with the use of advanced statistical techniques (e.g., machine learning), it may well be possible to predict with reasonable certainty who will die within the coming 5 years. We believe this is an interesting and important direction for further research. The extent to which the third precondition is fulfilled depends on the specific institutional characteristics of the health insurance market. In the Dutch context, insurers face open enrollment and a standardized benefits package, making it impossible for them to refuse specific individuals or to refrain from contracting certain health care. Although in theory insurers could engage in risk selection via the customer service and/or the contracted provider network, it is doubtful whether that would be effective given that individuals who are near the end of life are likely to have a low propensity to switch health plans.

## Figures and Tables

**Figure A1.**
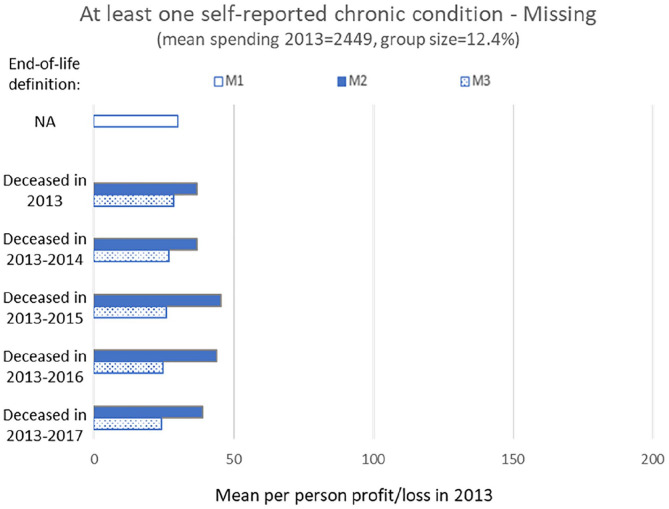
Mean Per-Person Profit in 2013 Under M1 to M3 for Five Definitions of Being Near the End of Life for the Group for Whom in 2012 the Information on Yes/No Self-Reported Chronic Condition Was Missing *Note.* M1 is the Dutch risk-equalization model 2016, M2 is M1 supplemented with 100% cost-based compensation for people who are near the end of life, and M3 is M1 supplemented with a proportional cost-based compensation using the percentages in the right column of Table 2. NA means not applicable. An asterisk (*) next to a set of bars indicates a statistically significant (*p* < .05) difference between the profit/loss under M2 versus M3.

## Appendices

### Appendix A

Mean per-person predictable profit/loss in 2013 under M1 to M3 for five definitions of being near the end of life for the group for whom in 2012 the information on yes/no self-reported chronic condition is missing.

### Appendix B

Mean per-person predictable loss in 2013 under three payment models, the absolute difference between M2 and M3 and the *t*-value for five definitions of being near the end of life for 19 chronic conditions individuals could report to have suffered from in the past 12 months.

**Table B1.** Mean Per-Person Loss in 2013 Under M1 to M3, the Absolute Difference Between M2 and M3 and the t-value for Five Definitions of Being Near the End of Life for 19 Chronic Conditions Individuals Could Report in 2012 to Have Suffered From in the Past 12 Months.

Groups	End-of-life definition	Mean per-person profit/loss under M1 (in Euros)	Mean per-person profit/loss under M2 (in Euros)	Mean per-person profit/loss under M3 (in Euros)	Absolute difference between M2 and M3. * indicates statistically significant difference (*p <* .05)	*T*-value of paired *t*-test between the mean per-person profit/loss under M2 versus M3
Stroke (*N* = 0.5%; mean spending = 9,093)	NA	−703	—	—	—	—
	Deceased in 2013	—	−781	−666	115	0.96
	Deceased in 2013–2014	—	−636	−629	7	0.05
	Deceased in 2013–2015	—	−535	−603	68	0.49
	Deceased 2013–2016	—	−474	−581	108	0.77
	Deceased in 2013–2017	—	−391	−562	172	1.21
Heart attack (*N* = 0.4%; mean spending = 8,504)	NA	−488	—	—	—	—
	Deceased in 2013	—	−637	−462	176	1.36
	Deceased in 2013–2014	—	−565	−437	128	0.85
	Deceased in 2013–2015	—	−544	−418	126	0.82
	Deceased 2013–2016	—	−599	−403	195	1.25
	Deceased in 2013–2017	—	−544	−390	154	0.84
Heart condition (*N* = 2.1%; mean spending = 8,919)	NA	−757	—	—	—	—
	Deceased in 2013	—	−659	−717	58	0.88
	Deceased in 2013–2014	—	−707	−678	29	0.42
	Deceased in 2013–2015	—	−627	−649	23	0.31
	Deceased 2013–2016	—	−746	−626	120	1.61
	Deceased in 2013–2017	—	−689	−605	84	1.11
Cancer (*N* = 1.7%; mean spending = 10,474)	NA	−1130	—	—	—	—
	Deceased in 2013	—	−632	−1070	438*	3.89
	Deceased in 2013–2014	—	−378	−1012	634*	5.29
	Deceased in 2013–2015	—	−340	−970	630*	5.31
	Deceased 2013–2016	—	−191	−934	744*	5.93
	Deceased in 2013–2017	—	−192	−904	712*	5.78
Migraine (*N* = 15.0%; mean spending = 2,307)	NA	−107	—	—	—	—
	Deceased in 2013	—	−97	−101	4	0.30
	Deceased in 2013–2014	—	−95	−96	0.3	0.02
	Deceased in 2013–2015	—	−95	−92	3	0.23
	Deceased 2013–2016	—	−104	−88	15	1.05
	Deceased in 2013–2017	—	−98	−85	13	0.85
Blood pressure (*N* = 16.0%; mean spending = 4,432)	NA	−214	—	—	—	—
	Deceased in 2013	—	−214	−202	11	0.74
	Deceased in 2013–2014	—	−200	−191	9	0.52
	Deceased in 2013–2015	—	−194	−183	11	0.59
	Deceased 2013–2016	—	−219	−177	43*	2.32
	Deceased in 2013–2017	—	−222	−171	51*	2.79
Blood vessels (*N* = 2.5%; mean spending = 7,604)	NA	−611	—	—	—	—
	Deceased in 2013	—	−568	−578	10	0.19
	Deceased in 2013–2014	—	−502	−547	45	0.76
	Deceased in 2013–2015	—	−364	−524	160*	2.55
	Deceased 2013–2016	—	−360	−505	145*	2.31
	Deceased in 2013–2017	—	−325	−489	163*	2.54
Asthma (*N* = 7.9%; mean spending = 4,732)	NA	−242	—	—	—	—
	Deceased in 2013	—	−185	−229	44	1.49
	Deceased in 2013–2014	—	−161	−217	56	1.72
	Deceased in 2013–2015	—	−183	−208	24	0.75
	Deceased 2013–2016	—	−180	−200	20	0.63
	Deceased in 2013–2017	—	−167	−194	26	0.80
Psoriasis (*N* = 2.7%; mean spending = 3,780)	NA	−386	—	—	—	—
	Deceased in 2013	—	−366	−365	0.2	0.00
	Deceased in 2013–2014	—	−374	−345	28	0.80
	Deceased in 2013–2015	—	−357	−331	26	0.71
	Deceased 2013–2016	—	−376	−319	57	1.54
	Deceased in 2013–2017	—	−345	−309	36	0.93
Eczema (*N* = 4.8%; mean spending = 2,790)	NA	−151	—	—	—	—
	Deceased in 2013	—	−133	−143	10	0.45
	Deceased in 2013–2014	—	−122	−136	14	0.56
	Deceased in 2013–2015	—	−107	−130	23	0.87
	Deceased 2013–2016	—	−120	−125	5	0.20
	Deceased in 2013–2017	—	−70	−121	51	1.85
Severe/recurring dizziness (*N* = 4.1%; mean spending = 5,819)	NA	−500	—	—	—	—
	Deceased in 2013	—	−484	−473	10	0.30
	Deceased in 2013–2014	—	−470	−447	22	0.58
	Deceased in 2013–2015	—	−432	−429	3	0.08
	Deceased 2013–2016	—	−465	−413	51	1.23
	Deceased in 2013–2017	—	−379	−400	20	0.47
Severe/recurring disease of intestines (*N* = 4.3%; mean spending = 5,285)	NA	−575	—	—	—	—
	Deceased in 2013	—	−603	−545	58	1.36
	Deceased in 2013–2014	—	−534	−515	19	0.42
	Deceased in 2013–2015	—	−512	−493	19	0.40
	Deceased 2013–2016	—	−515	−475	40	0.87
	Deceased in 2013–2017	—	−501	−460	41	0.89
Incontinence (*N* = 6.3%; mean spending = 5,630)	NA	−238	—	—	—	—
	Deceased in 2013	—	−299	−225	74*	2.99
	Deceased in 2013–2014	—	−262	−213	49	1.65
	Deceased in 2013–2015	—	−228	−204	23	0.75
	Deceased 2013–2016	—	−251	−197	54	1.71
	Deceased in 2013–2017	—	−218	−190	28	0.84
Wear of joint (*N* = 13.2%; mean spending = 4,870)	NA	−255	—	—	—	—
	Deceased in 2013	—	−262	−241	21	1.19
	Deceased in 2013–2014	—	−255	−228	27	1.38
	Deceased in 2013–2015	—	−227	−219	8	0.42
	Deceased 2013–2016	—	−239	−211	28	1.40
	Deceased in 2013–2017	—	−253	−204	49*	2.44
Joint inflammation (*N* = 5.0%; mean spending = 5,786)	NA	−334	—	—	—	—
	Deceased in 2013	—	−393	−317	76*	2.25
	Deceased in 2013–2014	—	−393	−299	94*	2.55
	Deceased in 2013–2015	—	−369	−287	82*	2.22
	Deceased 2013–2016	—	−376	−276	100*	2.69
	Deceased in 2013–2017	—	−373	−267	106*	2.82
Severe/recurring condition of back (*N* = 9.9%; mean spending = 4,016)	NA	−232	—	—	—	—
	Deceased in 2013	—	−219	−219	0	0.01
	Deceased in 2013–2014	—	−207	−207	0	0.00
	Deceased in 2013–2015	—	−212	−199	14	0.57
	Deceased 2013–2016	—	−245	−191	53*	2.25
	Deceased in 2013–2017	—	−223	−185	38	1.56
Severe/recurring condition of neck (*N* = 9.4%; mean spending = 3,731)	NA	−166	—	—	—	—
	Deceased in 2013	—	−137	−158	20	0.92
	Deceased in 2013–2014	—	−141	−149	8	0.37
	Deceased in 2013–2015	—	−145	−143	2	0.11
	Deceased 2013–2016	—	−171	−138	33	1.45
	Deceased in 2013–2017	—	−162	−133	29	1.26
Severe/recurring condition of elbow (*N* = 6.3%; mean spending = 4,385)	NA	−167	—	—	—	—
	Deceased in 2013	—	−133	−159	25	0.85
	Deceased in 2013–2014	—	−161	−150	11	0.36
	Deceased in 2013–2015	—	−139	−144	5	0.14
	Deceased 2013–2016	—	−180	−138	41	1.29
	Deceased in 2013–2017	—	−177	−134	44	1.33
Other (*N* = 13.8%; mean spending = 4,889)	NA	−377	—	—	—	—
	Deceased in 2013	—	−337	−357	20	0.97
	Deceased in 2013–2014	—	−311	−338	27	1.15
	Deceased in 2013–2015	—	−301	−324	23	0.95
	Deceased 2013–2016	—	−301	−312	11	0.44
	Deceased in 2013–2017	—	−289	−302	13	0.50

*Note.* M1 is the Dutch risk-equalization model of 2016, M2 is M1 supplemented with 100% cost-based compensation for people who are near the end of life, and M3 is M1 supplemented with proportional cost-based compensation using the percentages in the right column of Table 2. Comparing the results for individuals who reported to have suffered a stroke in the past 12 months with the result for those who reported to have ever suffered a stroke (Figure 3) shows a statistically significant difference between M2 and M3 for the latter group, but not for the first. The reason for this can be found in a complex interplay of three factors: (a) the difference in mean spending of these groups in year *t*, (b) the difference in mean risk-equalization payment of these groups in year *t*, and (c) the difference between these groups in terms of correlation with “being near the end of life.” In particular, the second factor might be relevant here since people who have suffered a stroke in the past 12 months are more likely to be flagged by a morbidity indicator (e.g., due to hospital treatment in year *t* − 1) than those who have suffered a stroke ever in the past.

## Notes

1. Van Vliet and Lamers (1998) showed that the above-average spending of decedents starts increasing 6 years prior to death, with the increase becoming steeper as people are closer to death.

2. Information on whether someone had actually died was not available for the rebalancing procedure, so we had to resort to a proxy.

3. Because the risk-equalization model is estimated by OLS, mean spending equals mean predicted spending in the estimation data. However, this is not necessarily the case for samples drawn from these data. Therefore, after the rebalancing but before calculating the group-level predictable profits and losses, we corrected the mean spending under each model estimation in the survey sample such that the overall mean spending and the overall mean predicted spending under each model are equal. We did this by multiplying individual-level spending by a factor obtained from dividing the mean predicted spending by the mean spending. These factors are 1.00246 for the base model and 1.00575, 1.0062, 1.00787, 1.0067, and 1.00693, respectively, for each of the definitions of end-of-life.

4. In this study, our primary focus is not on the predictability of death itself, but on the extent to which end-of-life spending contributes to group-level predictable profits and losses.
